# Housekeeping Gene Expression in the Fetal and Neonatal Murine Thymus Following Coxsackievirus B4 Infection

**DOI:** 10.3390/genes11030279

**Published:** 2020-03-05

**Authors:** Aymen Halouani, Habib Jmii, Hélène Michaux, Chantal Renard, Henri Martens, Dimitri Pirottin, Maha Mastouri, Mahjoub Aouni, Vincent Geenen, Hela Jaïdane

**Affiliations:** 1 Faculté de Pharmacie de Monastir, Université de Monastir, Laboratoire des Maladies Transmissibles et Substances Biologiquement Actives LR99ES27, Monastir 5000, Tunisia; halouani.aymen@yahoo.com (A.H.); jmiihbib@yahoo.fr (H.J.); mastourimaha@yahoo.fr (M.M.); aouni_mahjoub2005@yahoo.fr (M.A.); 2 Faculté des Sciences de Tunis, Université de Tunis El Manar, Tunis 1068, Tunisia; 3 Faculté de Médicine, Université de Liège, GIGA-I3 Immunoendocrinologie, CHU-B34, B-4000 Liege, Sart Tilman, Belgium; hmichaux88@gmail.com (H.M.); ch.renard@chu.ulg.ac.be (C.R.); hmartens@ulg.ac.be (H.M.); vgeenen@uliege.be (V.G.); 4 University of Liège, GIGA-I3 and Department of Functional Sciences, Laboratory of Cellular and Molecular Immunology, CHU-B34, B-4000 Liège, Sart Tilman, Belgium; dimitri.pirottin@uliege.be

**Keywords:** housekeeping genes, thymus development, thymic epithelial cells, swiss albino mice, in utero infection, Coxsackievirus B4

## Abstract

The thymus fulfills the role of T-cell production and differentiation. Studying transcription factors and genes involved in T-cell differentiation and maturation during the fetal and neonatal periods is very important. Nevertheless, no studies to date have been interested in evaluating the expressions of housekeeping genes as internal controls to assess the varying expressions of different genes inside this tissue during that period or in the context of viral infection. Thus, we evaluated by real-time quantitative polymerase chain reaction (qPCR) the expression of the most common internal control genes in the thymus of Swiss albino mice during the fetal and neonatal period, and following in utero infection with Coxsackievirus B4. The stability of expression of these reference genes in different samples was investigated using the geNorm application. Results demonstrated that the expression stability varied greatly between genes. *Oaz1* was found to have the highest stability in different stages of development, as well as following Coxsackievirus B4 infection. The current study clearly demonstrated that *Oaz1*, with very stable expression levels that outperformed other tested housekeeping genes, could be used as a reference gene in the thymus and thymic epithelial cells during development and following Coxsackievirus B4 infection.

## 1. Introduction

Coxsackievirus B (CV-B) group belongs to the human enterovirus B species, from the Enterovirus genus and Picornaviridae family. The virus particle is small, non-enveloped, and consists of a single-stranded RNA genome with positive polarity, encompassed in an icosahedral capsid. The genome comprises a single open-reading frame (ORF) flanked by two untranslated regions at both 5′ and 3′ ends, with a virus-encoded protein, VPg, linked to its 5′ terminus and a polyadenylated tail at its 3′ terminus. The ORF codes for a polyprotein that is proteolytically processed into 11 mature proteins, 4 of which are structural and 7 of which are non-structural (reviewed in Reference [1]).

CV-Bs cause both acute and chronic diseases. They have been implicated in the pathogenesis of aseptic meningitis, pancreatitis, type 1 diabetes (T1D), myocarditis, a serious disease that can lead to dilated cardiomyopathy, and cardiac failure (reviewed in Reference [2]).

At the molecular level, pathogenesis can involve a decrease in the expression of genes implicated in the immunity response and/or an increase in the expression of genes coding for pro-inflammatory cytokines which can lead to inflammation via the activation of mononuclear cells in the target organ during infection and, probably, to an early autoimmune response [3].

Among such targeted organs, a few studies carried out in vitro, ex vivo, and in vivo have explored the effect of thymic CV-B infection on the expression of different genes potentially involved in the autoimmune pathogenic process.

A persistent replication of CV-B4 E2 (diabetogenic) and JVB (prototype) strain in primary cultures of human thymic epithelial cells (TEC) has been demonstrated with an increased rate of TEC proliferation and with an increase in the secretion of the cytokines IL-6, LIF, and GM-CSF in the supernatants [4]. An upregulation of MHC class I was also observed following CV-B4 E2 infection of human fetal thymic organ cultures [5]. This MHC class I upregulation of thymic T cells and TEC was correlated with viral load and infectious particles. 

In a study conducted by Jaïdane and colleagues, it was shown that some enterovirus strains (CV-B4 E2 and JVB, CV-B3, and echovirus 1) were also able to infect a murine TEC line [6]. The infection induced a dramatic decrease in Igf2 transcripts and IGF-2 production in long-term cultures of this cell line.

Real-time quantitative PCR (qPCR) has become a cornerstone of molecular biology, and qPCR is currently the standard method used for the sensitive and specific detection and quantification of selected genes [7].

By determining the copy number (absolute quantification) or the relative expression, qPCR allows understanding of the different signaling and metabolic pathways that underlie variations during development and in response to different environmental stimuli. It is used for many purposes, including gene expression (mRNA) profiling analysis, genetic variation analysis and mutation detection, microRNA and noncoding RNA analysis, disease diagnosis, and also in cancer, drug, and food research (reviewed in Reference [8]).

Nevertheless, appropriate normalization is essential for obtaining an accurate, reliable, and robust measure of gene expression levels which allows correction of the variability resulting from different steps of the experimental procedure, such as RNA recovery and purity during extraction, RNA integrity, and efficiency of cDNA synthesis during reverse transcription (RT). The use of internal controls, housekeeping (HKGs) or reference genes, constitutes one of the normalization approaches; their expression is generally stable, since these genes are required for cellular survival. Notwithstanding, HKGs can undergo the same type of variations under experimental conditions, which requires validation of the stability of their expression in order to choose the most suitable candidate to use for normalization. In addition to alteration by experimental conditions, the expression level may vary from one individual to another and from one organ to another within the same body or during different developmental stages [9,10].

Although the thymus is most active during fetal and neonatal life, to the best of our knowledge, no study has evaluated the expression of HKGs during this period or following CV-B infection of that organ. In this work, we assessed the expression of different HKGs to validate the optimal control gene for the quantification of transcript levels during the development process of mouse thymus and following its in utero infection by CV-B4.

## 2. Materials and Methods

### 2.1. Virus

The CV-B4 E2 strain (kindly provided by J. W. Yoon, Julia McFarlane Diabetes Research Centre, Calgary, Alberta, Canada) was propagated in human epithelial type 2 (HEp-2) cells (BioWhittaker, Walkersville, MD, USA) in Eagle’s minimum essential medium (MEM; GIBCO BRL, Invitrogen, Gaithersburg, MD, USA) supplemented with 10% heat-inactivated fetal calf serum (FCS; GIBCO BRL), 1% (2 mM) L-glutamine (BioWhittaker), 1% non-essential amino-acids (Gibco BRL), 50 µg/mL streptomycin, 50 IU/mL penicillin (GIBCO BRL), and 0.05% (2.5 µg/mL) fungizone (Amphotericin B; Apothecon). Supernatants were collected 3 days post-inoculation (p.i.), clarified by centrifugation at 4000× *g* for 10 min, divided into aliquots, and stored at −80 °C. Virus titers in stocks were determined in HEp-2 cells by limiting dilution assay for 50% tissue culture infectious doses (TCID_50_), using the method of Reed and Muench [11].

### 2.2. Mice

Adult outbred Swiss albino mice (Pasteur Institute, Tunis, Tunisia) handled in the animal facility of the faculty of pharmacy of Monastir were used in this investigation. All experiments were conducted following the standards of general ethics guidelines and were approved by the Faculty of Pharmacy, University of Monastir, Tunisia. Animals used in this investigation were maintained in specific-pathogen-free conditions and allowed food and water ad libitum. Mice were mated (four females per male were caged together) until successful fertilization was noted. The day the vaginal plug was observed was considered the first day of gestation (day 1G).

### 2.3. Mouse Inoculation and Follow-Up

Pregnant mice were inoculated randomly, at either gestational Day 10 (10G) or 17 (17G), orally (by gavage) with 2 × 106 TCID50 of CV-B4 E2 contained in 300 µL of culture medium. Control animals were inoculated with 300 µL of sterile MEM. At each of different time points (Day 17G, and Days 1 and 5 from birth), mice were killed using isoflurane and offspring thymuses were sampled. All samples were washed with cold phosphate-buffered saline (PBS) and stored at −80 °C until RNA extraction.

### 2.4. TEC Isolation

Thymic lobes were isolated from all mice of the same litter for each group, washed in cold PBS and transferred into cold DMEM (Invitrogen) supplemented with 10% heat-inactivated FCS (Invitrogen), 20 mM HEPES, 50 µg/mL streptomycin, 50 IU/mL penicillin (GIBCO BRL), and 0.05% (2.5 µg/mL) fungizone (Amphotericin B; Apothecon). Thymuses were cut into small pieces and fragments were dispersed further via pipetting to remove the majority of thymocytes. After removing the supernatant, the resulting thymic fragments were subjected to two rounds of 30 min digestion with 125 µg/mL Liberase (Sigma-Aldrich) and 50 µg/mL DNase I (Roche Molecular Biochemicals) in DMEM at 37 °C for 15 min, with pipetting once in the middle of the incubation period. Then, supernatants were pooled and centrifuged, cell pellets were resuspended in 6 mL Percoll (1.07 g/mL), and DMEM was gently flowed over Percoll and centrifuged at 500× *g* for 30 min at +4 °C with the brake off. Interphase cells, which include antigen-presenting cells, were collected and, after adding 5 mL of DMEM 2% FCS, centrifuged at 500× *g* for 5 min at +4 °C. Cell pellets were resuspended in culture medium and were submitted to TEC enrichment using the MagniSort™ Mouse CD45 Positive Selection Kit (Invitrogen). Briefly, up to 10^8^ cells/100 µL were incubated with 20 μL of MagniSortTM Anti-Mouse CD45 Biotin B for 10 min at room temperature. After that, 4 mL of PBS (supplemented with 2% SVF, 1% penicillin, and streptomycin) was added and cells were washed by centrifugation at 500× *g* for 5 min. Cell pellets were resuspended and incubated for 10 min at room temperature with 20 μL of MagniSortTM Positive Selection Beads A. Up to 2.5 mL of cell separation buffer was added, and the tube was inserted into the 6-Tube Magnetic Separation Rack (Cell signal) and incubated for 5 min at room temperature. The supernatant containing CD45^-^ cells was collected and washed twice by centrifugation at 500× *g* for 5 min [12].

### 2.5. RNA Extraction

Total RNA was extracted using the NucleoSpin XS-kit (Macherey-Nagel, GmbH & Co, Düren, Germany) according to the manufacturer’s instructions. Briefly, thymuses were lysed in 102 µL of RA1-TCEP mixture. Five microliters of carrier RNA working solution were added to the lysate. The sample was filtered through the NucleoSpin® Filter Column. One hundred microliters of 70% ethanol was added to the lysate and RNA bound to the NucleoSpin® RNA XS Column. The column was desalted using membrane-desalting buffer. DNA was digested using DNase reaction mixture. rDNase was inactivated with RA2 buffer and silica membrane washed twice with RA3 buffer. Pure RNA was eluted in 10 µL of nuclease-free water (Ambion). The RNA concentration and quality were assessed using a NanoDrop 2000 (UV-Vis Spectrophotometer; ThermoScientific, Waltham, MA, USA).

### 2.6. cDNA Synthesis

RNA was reverse-transcribed using the Transcriptor First Strand cDNA Synthesis Kit (Roche). Complementary DNA (cDNA) synthesis was first performed with approximately 500 ng of RNA in a final volume of 20 µL containing 20 U/µL of Transcriptor Reverse Transcriptase, 2.5 µM of anchored-oligo(dT)18 primer, 60 µM of random hexamer primer, 20 U of RNase inhibitor (all contained in the kit), and 1 mM of each deoxynucleoside triphosphate (dNTP, Promega).

According to the manufacturer’s instructions, secondary structures were first denatured by heating the samples for 10 min at 65 °C in the presence of anchored-oligo(dT)18 primer, random hexamer primer, and nuclease-free water in 20 µL total volume. Tubes were then cooled on ice immediately, supplemented with buffer, protector RNase inhibitor, dNTP mix, and Transcriptor reverse transcriptase, and incubated for 10 min at 25 °C, followed by 30 min at 55 °C. The enzyme was inactivated by heating at 85 °C for 5 min, and the reaction was then stopped by placing tubes on ice. Two negative controls were performed in each reaction, one without RNA and the second without enzyme (RT minus control), to detect any genomic DNA contamination.

### 2.7. Quantitaive PCR with SYBR Green

qPCR was performed for seven genes using the iCycler iQ real-time detection system (Bio-Rad) using SYBR green detection. Primers (Eurogentec, Belgium) used for the PCR are listed in Table 1.

Each reaction consisted of 20 μL containing 1 μL of cDNA, 10 µL of 2× Takyon™ No Rox SYBR® MasterMix dTTP Blue, and 4 pmol of each primer.

The cycling conditions were 1 cycle of denaturation at 95 °C for 10 min followed by 40 three segment cycles of amplification (95 °C for 30 s, 60 °C for 30 s, 72 °C for 25 s), where the fluorescence was automatically measured during PCR. A melting curve was done for each primer pair to check the presence of one gene-specific peak and the absence of primer dimers.

The endpoint used in the qPCR, Ct, was defined as the PCR cycle number that crossed an arbitrarily placed signal threshold. All samples were amplified in triplicate and the means were used for further analysis. In the same way, two controls were also included in each PCR reaction, a reverse-transcription negative control (without reverse transcriptase) to confirm the absence of genomic DNA, and a non-template negative control to check for primer dimers.

### 2.8. Viral Load

One microliter of cDNA was used to quantify CV-B4 RNA by qPCR with the same program as described for HKGs. The primer sequences used were: forward primer EV1 5′-CAAGCACTTCTGTTTCCCCGG-3′ and reverse primer EV2 5′-ATTGTCACCATAAGCAGCCA-3′. Enterovirus 71 RNA control (Vircell, Kindly provided by Pr Didier Hober, Université Lille II) was used as a standard for a calibration curve containing five points ranging from 1.26 × 10^4^ to 1.26 copies/mL.

### 2.9. Analysis of Expression Stability

The amount of internal reference gene relative to a calibrator (fold change between two Ct values) has been expressed as fold difference, which was calculated as [20]

Fold difference = 2−ΔCt(1)

For each gene, the coefficient of variation (CV) of the cycle threshold (Ct) value was calculated. The CV equals the SD divided by the mean of Ct (expressed as a percentage). The MFC was calculated to reflect the minor variation in expression of those candidate HKGs within the large dataset. The MFC was defined as a ratio of the maximum and minimum values observed within the dataset.

GeNorm is the best algorithm used to determine the most stable HKG from a set of tested candidate genes in a given sample subjected to different experimental conditions [21]. geNorm was originally developed by Vandesompele et al. [21] and is implemented in qbase+ software (version 3.1, Ghent, Belgium). The geNorm stability M value can be determined by this software, in which every HKG is tested against the other genes in a pairwise variation that serially excludes the least stable genes from the analysis. 

A candidate HKG must have a small CV, MFC <2 [22], and the lowest M value [21]. The ranking of HKG was essentially based on the M value of geNorm (from lowest to highest), defined as the average pairwise variation for that gene with all the other tested control genes.

### 2.10. Statistical Analysis

Statistical analysis was performed with GraphPad Prism 5 software. The unpaired *t*-test was used for fold change analysis in CV-B4- versus mock-infected samples. A *p* value < 0.05 was considered statistically significant. * *p* value < 0.05, ** *p* value < 0.01, *** *p* value < 0.001, **** *p* value < 0.0001. Pearson’s correlation test was used to analyze the correlation between the viral load and the fold change of a given HKG at a given time point. Pearson’s *r* > 0: positive correlation; Pearson’s *r* < 0: negative correlation.

## 3. Results

### 3.1. Evaluation of RNA Purity, Integrity, and Contamination

High levels of RNA purity were obtained, as confirmed by A260/A280 ratios in the range of 1.8 to  2.0. RNA integrity was defined as an RNA Integrity Number (RIN) >8 by Agilent 2100 Bioanalyzer (Agilent technologies), and was revealed to be in the acceptable range for all samples. A single amplicon, represented by a generation of a single peak, for each candidate gene was confirmed by qPCR melting curve analysis. DNA contamination for all samples was ruled out by negative results (cycle threshold, Ct > 40) for RT (enzyme) minus control.

### 3.2. Housekeeping Gene Expression in the Developing Thymus

Seven HKGs commonly used in quantitative gene expression to normalize Ct values were used in this study, namely *Hprt*, *Actb*, *Sdha*, *RpS29*, *Rpl4*, *Oaz1*, and *Gapdh*, the transcripts of which were quantified using RT-qPCR.

Candidate reference genes showed Ct values between 15 and 27. The variability of selected HKGs in the thymus has been presented herein as standard deviations (SD) and ranges, expressed as an average fold change from the mean and as a maximum fold changes (MFC), respectively.

**Figure 1:**
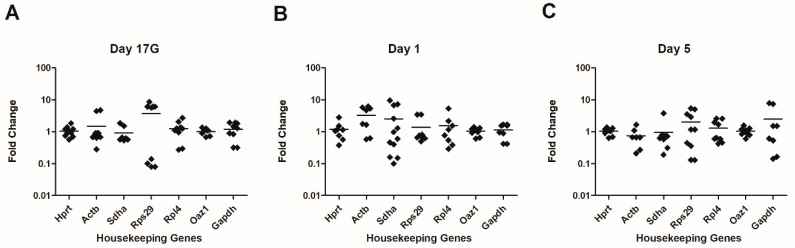
Fold change in HKG expression in the developing mouse thymus. mRNA was extracted and converted to cDNA. The cDNA was subjected to real-time quantitative PCR using gene-specific primers for seven selected HKGs. Expression variability of each HKG in thymuses (*n* = 7–12), sampled from fetuses at Day 17 of gestation (Day 17G) (**A**), and newborns at Day 1 (**B**) and Day 5 (**C**) from birth, is represented by log-scale of fold change (lines represent a median). The fold change represented was calculated using the equation presented by Livak and Schmittgen, 2001 [20]: Fold difference = 2−ΔCt.

As shown in Figure 1, *Oaz1* and *Hprt* were the most stable HKGs at the three tested time points. For *Oaz1*, the fold change was between 0.6 and 1.58 (average fold change = 1.033). For *Hprt*, the fold change was between 0.38 and 2.83 (average fold change = 1.087). *Rpl4* varied moderately, 2.5-fold at Day 17G and Day 5, and 5.3-fold at Day 1. There was considerably greater variability for *Actb* (up to 6-fold at Day 1), *Sdha* (up to 9-fold at Day 1), *RpS29* (up to 8-fold at Day 17G) and *Gapdh* (up to 7-fold at Day 5). Thus, we found *Oaz1* and *Hprt* to be the best combination of reference genes for studies of transcript levels in the developing murine thymus.

### 3.3. Housekeeping Genes Following CV-B4 Infection

The presence of CV-B4 E2 RNA in the thymus was checked at different p.i. times by RT-qPCR using specific primers of the highly conserved 5′ untranslated region of enterovirus genome, and infection was demonstrated in different samples. All samples retained for analysis of the effect of the infection on HKG expression were positive for CV-B4 RNA.

**Figure 2:**
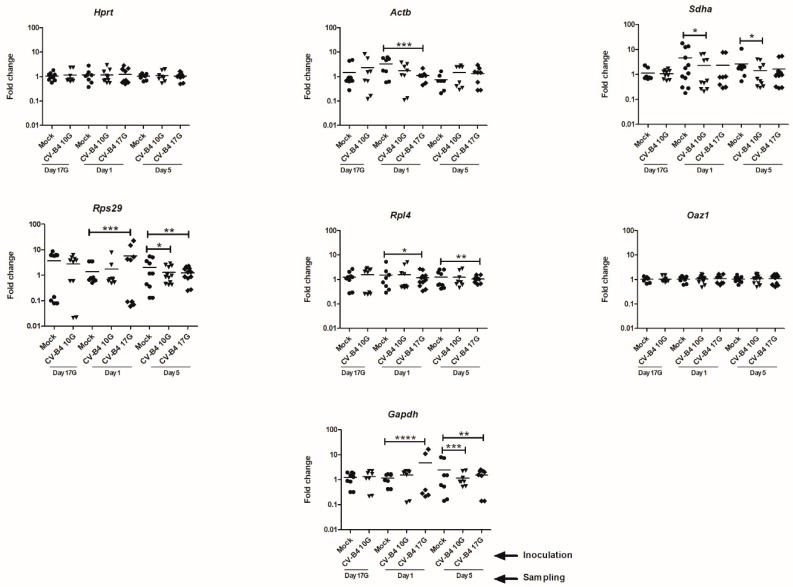
Fold change of seven candidate HKGs in CV-B4-infected compared to mock-infected thymus. Expression levels for each gene are represented by log-scale of fold change (lines represent a median) in mock- (●) and CV-B4-infected thymuses (*n* = 6–13), sampled at different time points, from fetuses at Day 17 of gestation (Day 17G), and newborns at Day 1 and Day 5 from birth. CV-B4 10G (▼): thymus sampled from mice born to dams inoculated with CV-B4 at Day 10 of gestation; CV-B4 17G (♦): thymus sampled from mice born to dams inoculated with CV-B4 at Day 17 of gestation. Variances in CV-B4- versus mock-infected samples were compared using the unpaired *t*-test. A *p* value < 0.05 was considered statistically significant. *p value < 0.05, ** *p* value < 0.01, *** *p* value < 0.001, **** *p* value < 0.0001.

Fold changes in HKG expression in CV-B4-infected compared to mock-infected thymuses, at different p.i. times, are represented in Figure 2.

*Oaz1* expression remained fairly stable from Day 17G to Day 5, even in infected thymuses, with fold changes between 0.5 and 1.7 following infection at Day 10G or 17G, as in mock-infected thymuses. *Hprt* seemed to be a little less stable, with fold changes between 0.37 and 2.92. A significant decrease in *Actb* was noted at Day 1 following CV-B4 inoculation at Day 17G (fold change between 0.45 and 2.2 versus 0.58 and 6.17 in mock-infected thymuses, *p* = 0.022). For *Sdha*, a significant variation was noted at Day 1 (fold change from 0.21 to 6.73 versus 0.18 to 17.75 in mock, *p* = 0.015) and Day 5 (fold change from 0.29 to 4.06 versus 0.54 to 10.72 in mock, *p* = 0.041) following CV-B4 inoculation at Day 10G. For *Rpl4*, a significant variation in expression was observed at Day 1 and Day 5 following CV-B4 inoculation at Day 17G (fold change from 0.369 to 2.71 versus 0.29 to 5.55 in mock, *p* = 0.012 and from 0.63 to 1.55 versus 0.42 to 2.58 in mock, *p* = 0.0098, respectively). A significant variation in *Gapdh* expression following CV-B4 inoculation at Day 17G was equally observed at Day 1 and Day 5 (fold change from 0.21 to 16.56 versus 0.42 to 1.67 in mock, *p* < 0.0001 and from 0.14 to 2.51 versus 0.14 to 7.93 in mock, *p* = 0.0036, respectively), together with a significant variation at Day 5 following CV-B4 inoculation at Day 10G (fold change from 0.51 to 2.36 versus 0.14 to 7.93 in mock, *p* = 0.0008). *RpS29* gene was the most variable gene, with variation at Day 1 (fold change between 0.06 and 22.45 versus 0.5 and 3.48 in mock, *p* = 0.0001) consequent to virus inoculation at Day 17G, and significant variations at Day 5 following CV-B4 inoculation at either Day 10G or 17G (fold change from 0.42 to 2.93 and from 0.25 to 2.28 versus 0.13 to 5.39 in mock, *p* = 0.017 and *p* = 0.0026, respectively).

**Figure 3:**
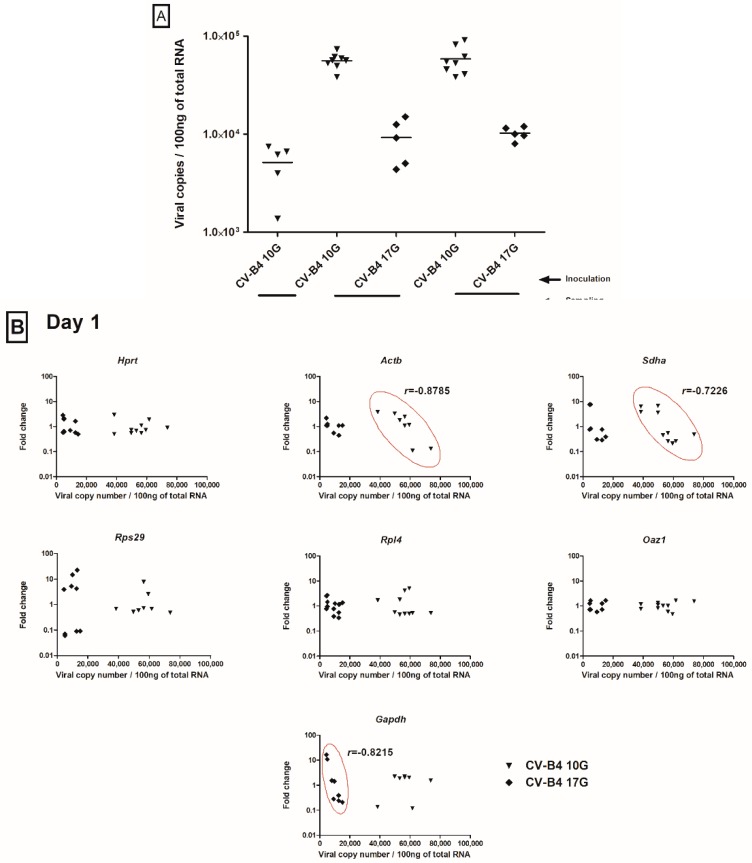

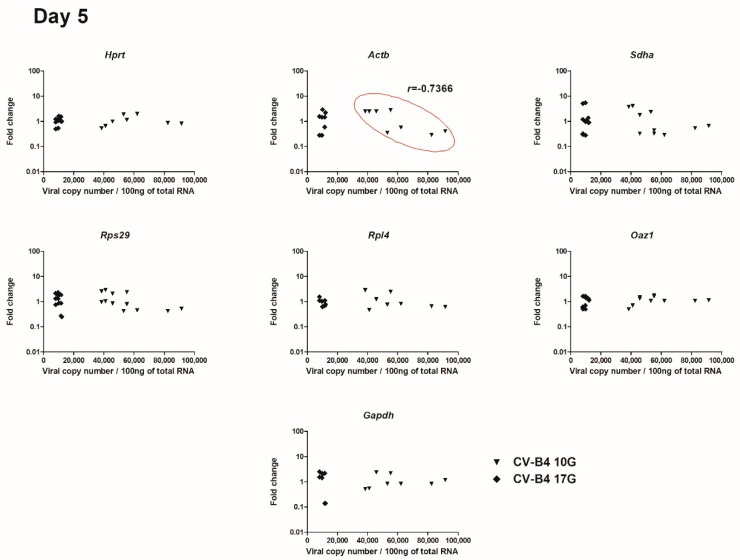
Effect of CV-B4 viral load on HKG expression. (**A**). Viral load in infected thymuses. Thymuses (*n* = 6–13) were sampled from fetuses at Day 17 of gestation (Day 17G) and from newborns at Day 1 and Day 5 from birth, following an infection at Day 10 or 17 of gestation (Day 10G ▼ or 17G♦). Total RNA was extracted, converted to cDNA, and the level of viral RNA in the thymus was determined by qPCR and is expressed here as copy number per 100 ng of total RNA. (**B**). Relationship between viral load and fold change in HKG expression in the infected thymus. For each sampled thymus, both fold change in HKG expression and the corresponding viral load were measured and plotted in the same graph. To better assess the effect of infection on HKG expression, a possible correlation between both parameters was evaluated using Pearson’s correlation test. Elliptic forms show negative correlations. r is the Pearson correlation coefficient. Only results for thymuses sampled at Days 1 and 5 from birth are represented here, since those sampled from fetuses at Day 17G showed neither effect of the infection (Figure 2) nor evident correlation with the viral load. CV-B4 10G (▼): thymus sampled from mice born to dams inoculated with CV-B4 at Day 10 of gestation; CV-B4 17G (♦): thymus sampled from mice born to dams inoculated with CV-B4 at Day 17 of gestation.

To better assess the effect of CV-B4 infection on HKG expression, the intensity of the infection was evaluated through measurement of the viral RNA copy number in the different samples. As illustrated in Figure 3A, viral loads could be segregated into two levels: low loads (around 8000 viral RNA copies/100ng of total RNA at Day 17G, following CV-B4 inoculation at day 10G, as well as at Days 1 and 5, following inoculation at Day 17G) and high loads (around 50,000 viral RNA copies/100 ng of total RNA at Days 1 and 5, following inoculation at Day 10G). Thus, at a given time point (mainly Day 1 or Day 5, because of the above-mentioned effects of the infection at those time points, as well as the marked differences in viral loads), we could appreciate an eventual correlation between the viral load and fold changes of the different HKG. As illustrated in Figure 3B, there were only few inverse correlations (Pearson’s *r* < 0) between the viral load and the fold change of a given HKG. In thymuses sampled the day of birth, this was the case for *Actb* and *Sdha* following CV-B4 inoculation at Day 10G (*r* = −0.8785 and *r* = −0.7226 respectively), and for *Gapdh* following CV-B4 inoculation at Day 17G (*r* = −0.8215). At day 5 from birth, only a negative correlation between the viral load and the fold change of *Actb* expression was observed in thymuses from mice inoculated at Day 10G (*r* = −0.7366). There was no correlation when, at a given time point, analyzing together samples from mice inoculated at Day 10G (high viral loads) and those inoculated at Day 17G (low viral loads). Any evident correlation could be found in thymuses sampled at Day 17G (data not illustrated).

### 3.4. Oaz1 as a Candidate Gene

A candidate HKG is defined as a gene with minimal variability in a tissue under different experimental conditions. It must have a small coefficient of variation (CV) and a MFC < 2.

All these parameters were calculated (Table 2) in all samples together, regardless of infection conditions and developmental stages. Tested HKGs were ranked from the most to the least stable according to different parameters (Mean Ct, SD, CV, MFC, and M value).

Taken together, all values within the dataset showed that *Oaz1* was the most stable among all seven tested HKGs, with the lowest CV, MFC, and M value (M = 1.665, calculated by geNorm).

*Hprt* and *Sdha* varied moderately with CVs equal to 5 and 6.9%, respectively, MFCs equal to 1.2 and M values equal to 1.7. *Actb*, *Rpl4*, *Gapdh*, and especially *RpS29* varied extremely, with CV > 12%, MFC > 1.6, and M value > 2 for *RpS29*.

Similarly, for TEC analysis (Table 3), *Oaz1* and *Hprt* had the lowest CV, MFC, and M values (1.308 for both genes), followed by *Gapdh* and *Sdha* which varied moderately with M value, equal to 1.693 and 1.839 respectively. For *Rpl4, Actb*, and *RpS29*, there was an extreme variation in expression, with the biggest CVs and MFCs and with an M value > 2.

## 4. Discussion

Real-time qPCR is a rapid, sensitive, and reliable method for the detection and quantification of mRNA transcription level. The principle of real-time PCR quantification is based on two concepts with different bases: absolute and relative quantification [7]. Absolute quantification determines the input copy number based on standard dilutions of known concentration. In relative or comparative quantification, the concentration of the target gene is expressed in comparison to a reference gene in the same sample.

HKGs are used as internal standards to normalize mRNA levels between different samples for a reliable comparison of mRNA transcription level. Although many studies have used those classic constitutive genes as presumed stable references, their expression may vary considerably depending on tissues, developmental stages, and specific experimental conditions [23]. It is therefore important to use as an internal control an HKG of which the expression remains stable during the studied growth and experimental conditions.

Until now, no validated reference genes have been reported for the normalization of transcript levels in the thymus during development or following CV-B infection. It is obvious that selection of a reference gene of high quality is of crucial importance for normalization, and must be identified with increasingly stringent standards. Thus, our aim was to identify an HKG with minimal variability under our different experimental conditions. 

For this purpose, a set of seven genes frequently used and validated (during development or under different experimental conditions in study conducted on humans or mice) as controls were chosen in our study, namely, *Hprt*, *Actb*, *Sdha*, *RpS29*, *Rpl4*, *Oaz1*, and *Gapdh*. We purposely chose genes with different functions (Table 1) in order to avoid genes belonging to the same biological pathway and that might be co-regulated.

First of all, gene expression was evaluated in thymuses (minimum *n* = 7) sampled from Swiss albino mice fetuses at Day 17G and neonates at Day 1 and Day 5. cDNA was obtained by reverse transcription of extracted RNA, and then quantified by qPCR.

Given the small size of the thymus during the fetal and neonatal stages, in order to obtain a pure RNA extract, total RNA was extracted by the NucleoSpin XS-kit since it seemed to be the most effective method for the extraction of high-quality RNA [24]. RNA integrity and purity was verified by RIN and the ratio A260 nm/A280 nm absorbance. The absence of DNA contamination was checked by a single peak qPCR analysis, Ct > 40 for RT minus control. Obtaining a unique qPCR product on gel electrophoresis confirmed the absence of primer dimers.

To determine the most stable gene, a combination between CV, MFC, and M value (calculated by geNorm) parameters was used. Our study demonstrated that the expression stability varied greatly between genes, and the expressions of six out of the seven investigated genes were found to be highly variable.

Variation in the expression of some HKGs during cell proliferation and differentiation has already been described. Furthermore, Piechaczyk et al. [25] found that *Gapdh* mRNA abundance varies from rat tissue to another. Similarly, an elevated expression of *Hprt* mRNA in the central nervous system, compared to other tissues, has been reported [26].

The combination of *RpS29*, *Rpl4*, and *Oaz1* was used in a study conducted by Taves et al. [27] to evaluate genes implicated in the glucocorticoid metabolic pathway in lymphoid organs, without addressing the issue of expression stability. In contrast to our study, the evaluation of gene expression using those three HKGs was in the thymus of neonatal (6 days old) and adult C57BL/6 mice.

Among one subset of 535 genes expressed in 11 major fetal and adult human tissues, only 47 transcripts were expressed at the same level in fetal and adult tissues [28]. An increase in *Gapdh* expression was also noted during fetal rat brain development [29].

Our current results showed that *Oaz1* was the most stable among all seven tested candidate genes in fetal and neonatal Swiss albino mice thymus. 

Recently, Medrano and associates [13] demonstrated that, in the thymus, among six tested genes, *Tbp* (TATA box binding protein) gene was the most stable gene during the period, versus *Hprt1* during the post-natal period (Day 21). Contrary to our current study, that investigation was performed on inbred mice (C57BL/6J strain) and only in the context of development, without addressing the infectious context.

Compared to the thymus, HKG expression in isolated TECs did not present a big difference. *Oaz1*, *Hprt*, then *Gapdh* had the smallest variations and *RpS29* was the least stable gene. Similarly, in a study conducted by St-Pierre et al. [30], whole-transcriptome sequencing revealed that *Oaz1*, *Hprt*, and *Gapdh* presented the same transcript abundance in cTECs and mTECs isolated from the thymus of 7 day old C57BL/6 mice.

Kermani and colleagues [31] evaluated the expression of the growth-hormone- and insulin-related antigens in Balb/c thymus during the embryonic and neonatal period. Although they chose *Hprt* as an internal gene in their study, the question of instability of that reference gene was not addressed. In the current study, we worked with a different mouse strain, namely Swiss albino mice, and in an infectious context.

Despite the scarcity of investigations, instability in HKG expression following an infection has been previously demonstrated. Furthermore, an increase in ribosomal RNA (rRNA) expression was reported following cytomegalovirus (CMV) infection of human fibroblast cells [32]. An extreme variation in *Actb* and *Gapdh* was found during Herpes Simplex Virus-1 (HSV-1) infection of human embryonic lung fibroblasts [33]. In a study conducted by Watson and co-workers [34], among 10 tested reference genes, only 3 (Peptidylprolyl isomerase A, *Gapdh*, and *Sdha*) showed any variation in cell cultures infected by human immunodeficiency virus type 1 (HIV-1), HSV-1, CMV, and varicella zoster virus (VZV). In the current study, CV-B4 infection had a significant effect on the expression of *Gapdh* and *RpS29*, then *Actb* and Rp14, then *Sdha*. These genes were affected by either increase and/or decrease of their expression, observed at Day 1 and/or Day 5 from birth, and mainly following CV-B4 inoculation at Day 17G but also following infection at Day 10G. Plotting fold changes with the corresponding viral loads in an attempt to better assess the effect of the infection revealed only a few negative (inverse) correlations among samples showing a significant effect of the virus (one with *Sdha* and the other with *Gapdh*). A negative correlation was also observed between the viral load following inoculation at Day 10G and the corresponding fold change of *Actb* (at both Day 1 and Day 5 from birth), although a significant effect of the virus on *Actb* expression could be observed only following inoculation at Day 17G. A more important variation was observed following mouse inoculation at Day 10G. This pronounced effect may logically be attributed to the higher viral load in thymuses sampled following an inoculation at Day 10G than in those sampled following inoculation at Day 17G.

During development, a gene’s expression is subject to multiple factors that cause it to vary. In the context of viral infection, intracellular replication and protein expression of CV-B may interfere with cellular physiology. In general, the virus diverts cellular machinery in its favor. Ribosomal genes are among those that may be affected. *Rps20* gene expression was noted in a previous study following CV-B3 infection of rat cardiac myocytes [35]. Nevertheless, no previous study has demonstrated an effect of CV-B on such genes. As described in the introduction, CV-B preferentially infects the pancreas, heart, and central nervous system, and thus, studies have generally focused on the effect of the virus on the expression of specific genes of those tissues or of genes involved in the immune response.

Other conditions and treatments can also affect HKG expression, which necessitates their validation as internal reference genes before use. Indeed, HKG expression has been reported to vary considerably in leukemic patients’ tumor samples [36] and in glioblastoma samples [37]. Additionally, the expression of *Gapdh* mRNA increases in case of insulin stimulation [38]. Furthermore, Burger and co-workers [39] demonstrated that chemotherapeutic drugs inhibit rRNA synthesis at different levels.

According to our knowledge, this work is the first validation of HKG in the thymus and isolated TECs of Swiss albino mice following in utero infection with Coxsackievirus B4, and provides useful data supporting further gene analysis in thymus mice during the fetal and neonatal period.

## 5. Conclusions

In conclusion, based on both expression stability and expression level, our data suggest that *Oaz1* is a good reference gene for evaluation and normalization of gene expression in the thymus during ontogeny and after CV-B4 infection. To our best knowledge, this was the first study to assess the expression stability of candidate reference genes in the thymus during fetal development (Day 17G), in neonatal life (Day 1 and 5), and after in utero viral infection.

## Figures and Tables

**Table 1:** Primer sequences of housekeeping genes used in qPCR.

Gene Symbol	Name	Function	Sequences	Amplicon Length (Base Pairs)	Gene ID	References
***Hprt***	Hypoxanthine phosphoribosyltransferase	Purine synthesis in salvage pathway	F 5′-gattagcgatgatgaaccaggtt-3′ R 5′-cctcccatctccttcatgaca-3′	150	15452	[13]
***Actb***	beta-Actin	Cytoskeletal structural protein	F 5′-tggaatcctgtggcatccatg-3′ R 5′-taaaacgcagctcagtaacag-3′	349	11461	[14]
***Sdha***	Succinate dehydrogenase complex subunit A	Electron transporter in the Krebs cycle and respiratory chain	F 5′-ggaacactccaaaaacagacct-3′ R 5′-ccaccactgggtattgagtagaa-3′	106	66945	[15]
***RpS29***	Ribosomal protein S29	Structural constituent of ribosome	F 5′-ggtcaccagcagctctactg-3′ R 5′-gtccaacttaatgaagcctatgtcc-3′	165	20090	[16]
***Rpl4***	Ribosomal protein L4	Structural constituent of ribosome	F 5′-cttgccagctctcattctctg-3′ R 5′-tggtggttgaagataaggttga-3′	125	67891	[17]
***Oaz1***	Ornithine decarboxylase antizyme 1	Cell growth and proliferation by regulating intracellular polyamine levels	F 5′-gccaatgaacgagatcactt-3′ R 5′-gctgtttaagatggtcaggtga-3′	110	18245	[18]
***Gapdh***	Glyceraldehyde-3-phosphate dehydrogenase	Carbohydrate metabolism	F 5′-tcaacgggaagcccatca-3′ R 5′-ctcgtggttcacacccatca-3′	209	14433	[19]

**Table 2:** Ranking of the seven tested housekeeping genes in thymus samples, from the most to the least stable.

Gene	Mean Ct	SD	CV (%)	MFC	M Value
*Oaz1*	23.1	0.578	2.503	0.912	1.665
*Hprt*	24.8	1.265	5.098	1.254	1.745
*Sdha*	23.94	1.414	6.911	1.291	1.775
*Actb*	18.63	1.616	8.674	1.4	1.865
*Rpl4*	20.46	1.644	6.86	1.31	1.970
*Gapdh*	18.66	2.05	10.987	1.507	2
*RpS29*	19.2	2.48	12.91	1.625	2.22

**Table 3:** Ranking of the seven tested housekeeping genes in isolated TECs, from the most to the least stable.

Gene	Mean Ct	Moy 2^^-Δct^	SD	CV (%)	MFC	M Value
*Oaz1*	21.51	1.075	0.567	2.638	1.05	1.308
*Hprt*	22.94	1.455	1.379	6.011	1.111	1.308
*Gapdh*	18.43	1.63	1.76	9.584	1.17	1.693
*Sdha*	24.31	1.79	1.945	8	1.172	1.839
*Rpl4*	20.67	1.834	1.902	9.203	1.199	2.145
*Actb*	18.67	2.377	2.54	13.61	1.32	2.391
*RpS29*	19.38	3.931	3.624	18.7	1.455	2.817

## Abbreviations

*Actb*	beta-Actin
Ct	cycle threshold
CV	coefficient of variation
CV-B	Coxsackievirus B
*Gapdh*	Glyceraldehyde-3-phosphate dehydrogenase
HKG	housekeeping gene
*Hprt*	Hypoxanthine phosphoribosyltransferase
MFC	maximum fold change
*Oaz1*	Ornithine decarboxylase antizyme 1
ORF	open-reading frame
qPCR	real-time quantitative PCR
*Rpl4*	Ribosomal protein L4
*RpS29*	Ribosomal protein S29
RT	reverse transcription
SD	standard deviation
*Sdha*	Succinate dehydrogenase complex subunit A
T1D	Type 1 diabetes
TCID_50_	tissue culture infectious doses
TEC	thymic epithelial cells
